# Time matters: on the predictive power of current, short- and long-term expected valence in an experience based learning task

**DOI:** 10.3389/fpsyg.2025.1570369

**Published:** 2025-09-30

**Authors:** Daniel Thomas Jäger, Jascha Rüsseler

**Affiliations:** ^1^Department of Psychology, Cognition, Emotion and Neuropsychology, University of Bamberg, Bamberg, Germany; ^2^Bamberg Graduate School of Affective and Cognitive Sciences (BAGrACS), University of Bamberg, Bamberg, Germany

**Keywords:** current valence, expected valence, subjective value, loss aversion, framing effects, recurrent decisions

## Abstract

This study investigates the predictive power of current, short-, and long-term expected valence in decision-making within an experience-based learning task. Across two experiments participants engaged in a gambling task where they had to balance short- and long-term outcomes to maximize gains. In Experiment 1 participants had to accept short-term losses to achieve long-term gains, while in Experiment 2 they had to omit short-term gains. Results from generalized mixed-effects models revealed that all three valence constructs (current, short-term, and long-term expected valence) were significant predictors of risky choices, with their influence modulated by the specific choice context. In a loss context participants relied more on short-term expectations, while in an omission context long-term expectations played a stronger role. These findings align with existing literature on the influence of emotional valence on decision-making and demonstrate the adaptability of the subjective valuation system across different choice scenarios. The study highlights the importance of considering multiple emotional self-report dimensions in decision-making processes.

## Introduction

Emotions are an integral part of decision making (e.g., Bechara et al., 1997; Charpentier et al., 2016; Dunning et al., 2017; Jäger et al., 2020; Lerner et al., 2015; Mellers et al., 1999; Schlösser et al., 2016). Theories in the field of emotions and decision-making often provide broad conceptualizations of emotions. Hence, these models lack the necessary precision to fully account for the complexity of emotional involvement in decision-making. For example, the Somatic Marker Hypothesis (SMH; Bechara et al., 1997; Reimann and Bechara, 2010) posits that physiological arousal serves as a “marker” for emotional activation that guides decision making. Importantly, the SMH postulates that conscious cognitive processes, at least in some situations, do not guide decisions, and that decisions are guided by emotions tied to the decision object. This central claim has been challenged by studies showing a parallel development of verbalizable knowledge about task structure and skin conductance responses (index of emotional arousal) in the pre-decision phase (Maia and McClelland, 2004). While the SMH offers valuable insights into the role of emotion in decision-making, it does underestimate the role of (conscious or unconscious) cognitive processes in decision making. In contrast, Lerner et al.'s (2015, 2023) framework provides a more detailed conceptualization by distinguishing between current and expected emotions. Nevertheless, this distinction does not sufficiently capture the variability of emotional expectations across different timeframes. Lerner’s model treats expected emotion as a singular construct, but it does not differentiate between short-term and long-term expectations, which have been shown to have distinct effects on decision-making (e.g., Ariely and Loewenstein, 2000; Seaman et al., 2022).

Hence, disagreement still exists on how to best conceptualize the different self reported emotion types that are involved in the decision process (Lerner et al., 2015; Loewenstein and Lerner, 2003; Västfjäll and Slovic, 2013). It has been proposed to separate *predecisional* from *postdecisional* emotions (Västfjäll and Slovic, 2013). *Predecisional* emotion is present before the decision is made. *Postdecisional* emotion is present after the decision is made, which is also referred to as *outcome emotion*. However, there is disagreement on how to conceptualize predecisional emotional constructs (Lerner et al., 2015; Västfjäll and Slovic, 2013). One possibility is to distinguish between two main predecisional emotional concepts: *current emotion* and *expected emotion*. We define *current emotion* as the current self-reported feeling that incorporates both immediate background emotions and integral emotions (directly related to the decision task at hand), drawing on an average of all available emotional information while including past experiences and cognitive expectations (Efendić et al., 2020; Lerner et al., 2015). Although some propose distinguishing between background and integral emotions (Dunning et al., 2017), research suggests that the emotional system does not separate relevant from irrelevant information. Instead, current emotions summarize all present feelings, whether tied to the current decision or past experiences (Asutay et al., 2021). *Expected emotion* refers to the anticipation of future emotional consequences based on the available choice options. Expected short-term emotion refers to the emotions individuals expect experiencing as a result of an immediate outcome of a decision, such as the immediate pleasure of a win or the discomfort of a loss. This type of emotion is closely tied to the anticipation of an immediate gain or loss following a decision (for more details see Jäger et al., 2022). In contrast, expected long-term emotion refers to emotions anticipated in the future, often after a longer period of time, such as the end of an experiment or a long-term goal, where outcomes might include greater rewards or costs (Duckworth et al., 2007). Understanding how these different timeframes of expected emotions influence the decision making process allows for a better grasp of how people navigate the tension between immediate outcomes and future consequences in decision-making (e.g., Ariely and Loewenstein, 2000).

Self-reported emotions might be a window into the subjective valuation of choice options (Vollberg and Sander, 2024). There are two main group of theories of value-based choice: prospect theory (Kahneman, 1979) and reinforcement learning theories (Sutton and Barto, 1999). Both theories offer key insights into how individuals assess and compare the subjective value of different choice options. These models go beyond traditional rational approaches like expected utility theory, emphasizing that decision making is influenced not only by logical calculation but also by psychological and emotional factors. Prospect theory highlights how people evaluate potential outcomes as gains or losses in relation to a reference point, often showing an aversion to losses that outweighs their preference for equivalent gains (e.g., Mellers et al., 2021; Nabi et al., 2020; Prietzel, 2020). For instance, individuals tend to prefer a sure gain of €500 over a 50% chance to win €1,000, demonstrating risk aversion when it comes to gains. However, the same individuals might take risks to avoid a loss, such as preferring a 50% chance to lose €1,000 over a guaranteed loss of €500, reflecting risk-seeking behavior in the domain of losses. The theory’s value function is concave for gains, convex for losses, and steeper for losses. Reinforcement learning theories focus on how individuals learn to make decisions over time by using feedback from their environment. These theories describe how people adjust their behavior based on rewards and punishments gradually learning to maximize long-term gains by choosing options that have led to positive outcomes in the past. Reinforcement learning models often involve trial and error through which individuals estimate the value of each option and update their choices based on new experiences (Sutton and Barto, 1999). Both models highlight the role of subjective value in decision making, yet they emphasize different mechanisms: prospect theory focuses on the emotional biases that affect risk perception, while reinforcement learning explains how choices evolve through experience and feedback. Together, these models provide a good understanding of how people weigh their options and make decisions, especially in uncertain situations. However, how subjective value is determined and how emotions contribute to the development of subjective value remains to be specified.

To further explore how self-reported emotions influence decision-making, Charpentier et al. (2016) conducted an experiment to examine the relationship between people’s feelings and their choices. They used self-reported feelings to develop a “feeling function,” quantifying how emotions associated with different outcomes relate to objective value. The study tested whether this “feeling function” could predict participants’ choices in subsequent decision-making tasks, while exploring the symmetry of feelings toward gains and losses and their influence on risk-related decisions. The results revealed that the “feeling function” was concave for gains and convex for losses, similar to the value function in prospect theory. This curvature reflects diminishing sensitivity, meaning that the emotional impact of smaller gains or losses—such as winning or losing $10—was felt more intensely than that of larger amounts like $100. Surprisingly, the study found no inherent asymmetry between feelings related to gains and losses, challenging the assumption that losses evoke stronger emotions than equivalent gains. However, when it came to decision-making, participants gave more weight to their feelings about losses than to those about gains, aligning with loss aversion. This suggests that while gains and losses may evoke similar intensity of emotional responses, people focus more on potential losses when making decisions, leading to risk-averse behavior in mixed gamble scenarios.

In the present paper we are interested in the subjective valuation of choice options, which we hypothesize is reflected in self-reported emotion measures (Vollberg and Sander, 2024). Our goal is to model the role of feelings in a reinforcement model based on subjective emotions, as previous research has shown to be feasible (Hayes and Wedell, 2020). Specifically, we aim to investigate how emotional constructs at a given time point can predict subsequent choices. In other words, we intend to examine predecisional emotional constructs with respect to different time frames and assess their predictive power in influencing choice behavior. As an example, Schlösser et al. (2013) found that immediate feelings experienced at the moment of decision-making predict risky choices, even more so than anticipated emotions or subjective probabilities. This underlines the role of real-time emotion in shaping behavior, particularly in high-stakes scenarios where decisions may be influenced by emotional responses to the options themselves rather than consideration of potential outcomes. Expectancy approaches, in contrast, argue that expected emotions, such as expected regret or pleasure, are the key drivers of choice. Research by Mellers et al. (1999) demonstrated that individuals often base their decisions on forecasts of how they will feel after various outcomes. For example, people might avoid a risky gamble because they anticipate the regret they would experience after a potential loss, highlighting the importance of cognitive expectations of future emotional consequences. Interaction approaches suggest that both current and expected emotions work together to influence decision-making. Jäger et al. (2022) found that in recurrent decision tasks, both current and expected emotional valence interact to predict choice. Valence refers to the intrinsic pleasantness or unpleasantness of an emotional experience and is a fundamental dimension of emotional states (Colombetti and Kuppens, 2024; Russell, 1980). Furthermore, Jäger et al. (2022) showed that while expected valence had the strongest influence on decision-making, current valence and its interaction with expected valence still significantly contributed to the prediction of choice. Interestingly, participants relied more on current emotional states if their expectations were unclear or positive, indicating a dynamic relationship between the two types of predecisional emotions. These findings suggest that the interaction between current feelings and future expectations is essential for understanding how individuals make decisions, particularly in repeated or experience-based tasks.

Despite the valuable insights from these theories, there is still limited research into how the time horizons of expected emotion influence decision-making. Most studies do not thoroughly examine whether people’s decisions are better predicted by their expectations of immediate emotional reactions or long-term outcomes (Charpentier et al., 2016; Jäger et al., 2020, 2022; Mellers et al., 1999; Schlösser et al., 2013). This distinction is important because emotional reactions can shift over time—what seems immediately appealing might not result in long-term satisfaction, and decisions that seem painful initially could offer benefits later on (e.g., Jennison, 2004; Kopainsky et al., 2019; Mueller et al., 2017). Investigating different time horizons could help to clarify how short-term versus long-term emotional expectations shape decision-making strategies, providing a more nuanced understanding of how expected emotions influence decision behavior. We were curious if adding a time horizon to predecisional valence expectations would result in another predictor of choice.

Manipulating expectations is essential for understanding how individuals make decisions, especially when emotions and time horizons are involved. Research has shown that narratives can shift people’s focus (Morag and Loewenstein, 2024). Similarly, altering the presentation of future outcomes, such as revealing the structure of a task, can change decision-making behavior (Jäger et al., 2022). Studies also show that people underweight factors like duration in decision-making (Ariely and Loewenstein, 2000). A recent meta-analysis found no significant relationship between age and preferences for immediate versus delayed rewards (Seaman et al., 2022). Hence, by reframing task structures and highlighting long-term benefits, researchers can manipulate time horizons, helping to reveal how individuals balance short-term and long-term considerations in their choices. Therefore, we designed two experiments to fully capture the time frames of emotional expectations. In the first experiment, participants had to accept losses to achieve long-term wins, while in the second experiment, they had to omit points to maximize overall wins. These experiments were designed to examine how different self-reported emotional constructs—current valence, expected short-term valence, and expected long-term valence—predict choice under different contextual demands, such as point-loss versus point-omission. By manipulating these decision contexts, we aimed to investigate how current and expected emotions across different time frames influence decision-making under varying conditions.

## Experiment 1

In a gambling task similar to previous experiments (Jäger et al., 2020, 2022) participants could earn points over the course of the experiment. The five participants with the highest score each received a movie theater voucher of 20 euros. In the experiment we confronted participants with decisions where they had to accept a short-term loss to achieve the long-term goal of winning as many points as possible. Thus, short-term and long-term goals did not align any more. First, participants did the task without knowing the internal structure of the task. After that, we assessed current feelings, short-term expectations and long-term expectations prior to the respective choice. In the second round, participants received information about the task structure and the most beneficial strategy of accepting short-term losses to win more points over the course of the experiment. Knowing this, they performed the task a second time. Thereafter, we assessed the same emotional variables again.

Based on the idea that the predictive power of emotional variables depends on the particular situation and the lack of research concerning the predictive power of long-term emotional valence, we wanted to show four things:

1. Long-term expected valence is an additional predictor of human choice besides current valence and short-term expected valence. Hence, we expected main effects of long-term expected valence, short-term expected valence and current valence.
2. Insight into the task structure increases the predictive power of long-term expected valence, because the instruction focuses on long-term benefits. Thus, we expected an interaction between insight (first vs. second round) and long-term expected valence.
3. Insight into the task structure decreases the predictive power of short-term expected valence, because the instruction defines the short-term losses as no obstacle towards the overall goal. Thus, we expected an interaction between insight (first vs. second round) and short-term expected valence.
4. Insight into the task structure should not influence the predictive power of current valence. Hence, we expected no significant interaction between insight (first vs. second round) and current valence.

### Method

#### Participants

Sample size was determined by simulating data of a pilot study using the SIMR package in R (Green and MacLeod, 2016). Alpha was set at 0.05, interaction and main effect slopes for choice prediction were set at 0.75, which corresponds to a medium effect size (Jäger et al., 2020, 2022 found effects within this range). For 50 simulations, the power remained above 0.8 for all effects and indicated an optimal sample-size of 35 participants (*M*_age_ = 24.2 years, SD = 3.87; 34 identified as female; 30 right-handed; normal or corrected to normal vision). All participants were students at the University of Bamberg and received course credit for participation. As an additional incentive, the best five participants each gained 20 euros as a voucher for a local movie theater. All of them gave their written informed consent and were debriefed afterwards. The local ethics committee approved the study protocol.

#### Materials

The experiment consisted of two parts, which each had a Learning Phase and Predecisional Valence Questionnaire blocks. For stimulus presentation, we used the NBS Presentation software. For answer collection, we used a two-keyed Cedrus Response Box (RB-380). For a more detailed description of the gambling task’s trial structure see Jäger et al. (2020, 2022).

##### Gambling task

Starting with a balance of 500 points, the participants of the gambling task were instructed to earn as many points as possible. In each trial, one of three different symbols was presented, and participants had to decide whether to gamble or not. If a participant decided to pass, the score always remained unaffected (+/− 0). If a participant decided to gamble, they could either win or lose points (+/− 15 points) depending on constant winning probability pairings for each symbol. The fundamental objective of the learning phase was to acquire and consolidate the symbols´ probability pairings. Moreover, at the beginning of the experiment, the symbols are randomly distributed to a Cue Contingency (CC) that determines the winning probabilities of the symbols. Symbols with CCs 1 and 2 result negatively 90% of the time (i.e., −15 points). Symbols with CC 3, on the other hand, are positive 90% of the time (i.e., +15 points). Based on the CC, the symbols differ in probabilities of occurrence, which can be represented using the example of an urn model: Assuming an urn is filled with balls, 10 of these balls are labeled with CCs 2 and 3 each. At the same time, 15 balls are assigned to CC 1. While the number of balls labeled with CCs 2 and 3 in this “imaginary urn” remains constant, the number of balls with CC 1 changes as follows: if a participant decides to play the symbol, a ball is drawn from the urn (at least one ball always remains in the urn). This reduces the probability of occurrence. Conversely, if they decide not to play the symbol, an additional ball is added to the urn. Consequently, the probability of occurrence of this symbol increases whenever the negative consequence of −15 points is avoided. After several trials participants find themselves in a situation where they have to constantly avoid CC1 symbols. Whenever the negative consequence is accepted one ball is removed from the urn, and therefore, the probability of occurrence of this symbol diminishes while the occurrence probabilities of the other two symbols increase. This means, over the whole course of the experiment it is beneficial to accept the losses of the CC1 symbols to get more opportunities to win points with the other symbols.

##### Predecisional valence questionnaire task

In the questionnaire blocks, participants continued the gambling task. We measured self-reported predecisional valence using a digital questionnaire format. Each time a symbol was presented, participants rated their current, expected short-term, and expected long-term valence before making the decision. Using a Self-Assessment Manikin Scale (Bradley and Lang, 1994), they marked their individual position on the visual analog scale by moving the mouse. The computer recorded the chosen point in a value ranging from −255 for a very unpleasant feeling to +255 for a very pleasant feeling. Starting point was always in the middle of the scale. Overall, we recorded three different question perspectives of valence for each symbol. The first perspective asked participants to rate their current valence (“How do you feel seeing this symbol?”). The other two question perspectives referred to the expected valence: One in relation to short-term expectations (“Please imagine you decide to gamble. How will you feel after you received the outcome of your decision?”) and the other one to long-term expectations (“Please imagine you decide to gamble. How will you feel when you see your score at the end of the experiment?”). The presentation of these questions and the corresponding symbols were randomized. Participants did not receive immediate feedback after responding, they merely obtained aggregated feedback by receiving their current score after each block.

#### Procedure

In the course of the study, participants first received a brief introduction to the procedure from the experimenter. As part of this, participants gave their informed consent to participate in the study and completed a short questionnaire that collected demographic data. Following this, participants were encouraged to ask questions at any time if something was unclear, and they were informed that they could withdraw from the study at any time. Participants were naive to the aims of the study.

Instructions were presented in written form and explained by the experimenter if necessary. A brief practice session for the Gamble Task followed. In this session, each symbol was presented once. After each decision, feedback was provided that 0 points were won, regardless of which key participants pressed. Participants should not form opinions about the symbols at this stage.

Further practice trials followed to familiarize participants with the questionnaire. In this session, participants were asked to assess their current valence, their expected valence after the decision, and at the end of the study in relation to the symbol. In the practice session, three questions about two symbols each were asked. After these three questions about a symbol, participants had to decide again whether they wanted to gamble or not (“Do you want to gamble?”). In the questionnaire section they did not receive immediate feedback on whether they had won or lost points.

After the two practice sessions, six gambling rounds followed, each with 27 trials (a total of 162 trials). Only now did the game decisions contribute to the starting score of 500. After each round, participants were offered a short break, and their total point score was displayed. To conclude the first half of the study, the questionnaire followed. In three rounds, participants had to answer 12 trials each with 3 questions (36 trials, 108 questions). In one round, each of the three symbols appeared four times. After participants answered all three questions about a symbol, they were asked if they would gamble (“Do you want to gamble?”). Unlike during the Gambling Task, they did not receive immediate feedback on whether they had won or lost points. After each round, participants had the opportunity to take a short break, and their point score was displayed.

In the second part of the study, participants received new instructions related to one of the three symbols, revealing a suitable gambling strategy. The instruction on the screen revealed the inner logic of the gambling task. That means, participants were told that it is more beneficial to accept losses with symbol CC1 as this decreases its probability. At the same time, the probability of occurrence of the other positive symbol increases which gives the opportunity to gather more points. Participants could ask questions until they told the experimenter that they understood the inner logic of the gambling task. The procedure of the second part of the study was identical to the first part. At the end of both halves of the study, participants were asked to report their point score to the experimenter. Subsequently, an explanation of the purpose and content of the study took place.

##### Analysis plan

First, we checked if our experimental manipulation worked as intended (manipulation check). After that, we built a model that predicted individual choices based on the proposed valence ratings. We used jamovi for data analysis (The jamovi project, 2023) conducting mixed effects models to test our hypotheses. For each model, we first reduced the random effect structure until the null model converged. Thereafter, we included the fixed effects according to our hypotheses. Valence variables were standardized before we included them in the respective models. Significant t-values are Bonferroni corrected.

### Results

#### Manipulation check

##### Presentation probabilities in the learning blocks for CC1 symbol

**Figure 1:**
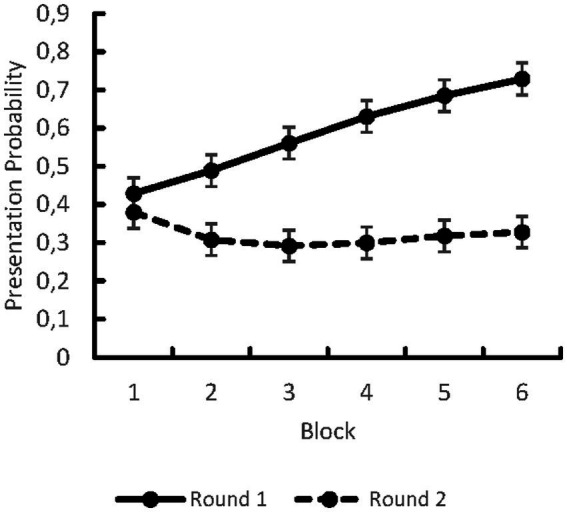
Presentation Probabilities for the CC1 symbol depending on Block and Round of the gambling task. Error-bars indicate Confidence Intervals.

We computed a linear mixed effects model for the presentation probabilities of the CC1 symbol. The described urn model determined presentation probabilities. Hence, for each trial we computed the presentation probability for CC1 symbols by the following formula: $(\mathrm{CC}1)=\frac{N_{\mathrm{CC}1}}{N_{\mathrm{CC}1}+N_{\mathrm{CC}2}+N_{\mathrm{CC}3}}$ , presentation probabilities for CC2 and CC3 symbols can be determined by the following formula: $P(\mathrm{CC}2)=\frac{1-P(\mathrm{CC}1)}{2}=P(\mathrm{CC}3)$ . We included the interaction of BLOCKxROUND and the main effects of BLOCK and ROUND as fixed effects in our model. The model converged including SUBJECT_ID as random effect, which resulted in the formula of CC1 PRESENTATION PROBABILITY ~ BLOCK*ROUND + (1|SUBJECT_ID). Estimates were fit by REML. There was a significant BLOCK*ROUND interaction, *F*(5, 11,294) = 391.0, *p* < 0.001, and two significant main effects: ROUND, *F*(1, 11,294) = 9592.0, *p* < 0.001, and BLOCK, *F*(5, 11,294) = 257.0, *p* < 0.001. The interaction revealed that in round 1, before participants had insight into the task structure, the mean presentation probabilities of CC1 symbols increased over the blocks, *M_Block1_* = 0.429, *CI* = [0.388, 0.471], *M_Block2_* = 0.489, *CI* = [0.448, 0.530], *M_Block3_* = 0.561, *CI* = [0.520, 0.603], *M_Block4_* = 0.631, *CI* = [0.589, 0.672], *M_Block5_* = 0.685, *CI* = [0.643, 0.726], *M_Block6_* = 0.729, *CI* = [0.688, 0.770]. However, in round 2, after participants had insight into the task structure, the mean presentation probabilities of CC1 symbols remained stable after an initial decrease, *M_Block1_* = 0.380, *CI* = [0.388, 0.471], *M_Block2_* = 0.308, *CI* = [0.267, 0.350], *M_Block3_* = 0.292, *CI* = [0.250, 0.333], *M_Block4_* = 0.300, *CI* = [0.259, 0.342], *M_Block5_* = 0.318, *CI* = [0.277, 0.360], *M_Block6_* = 0.328, *CI* = [0.287, 0.370]. See also Figure 1.

##### Valence ratings

**Figure 2:**
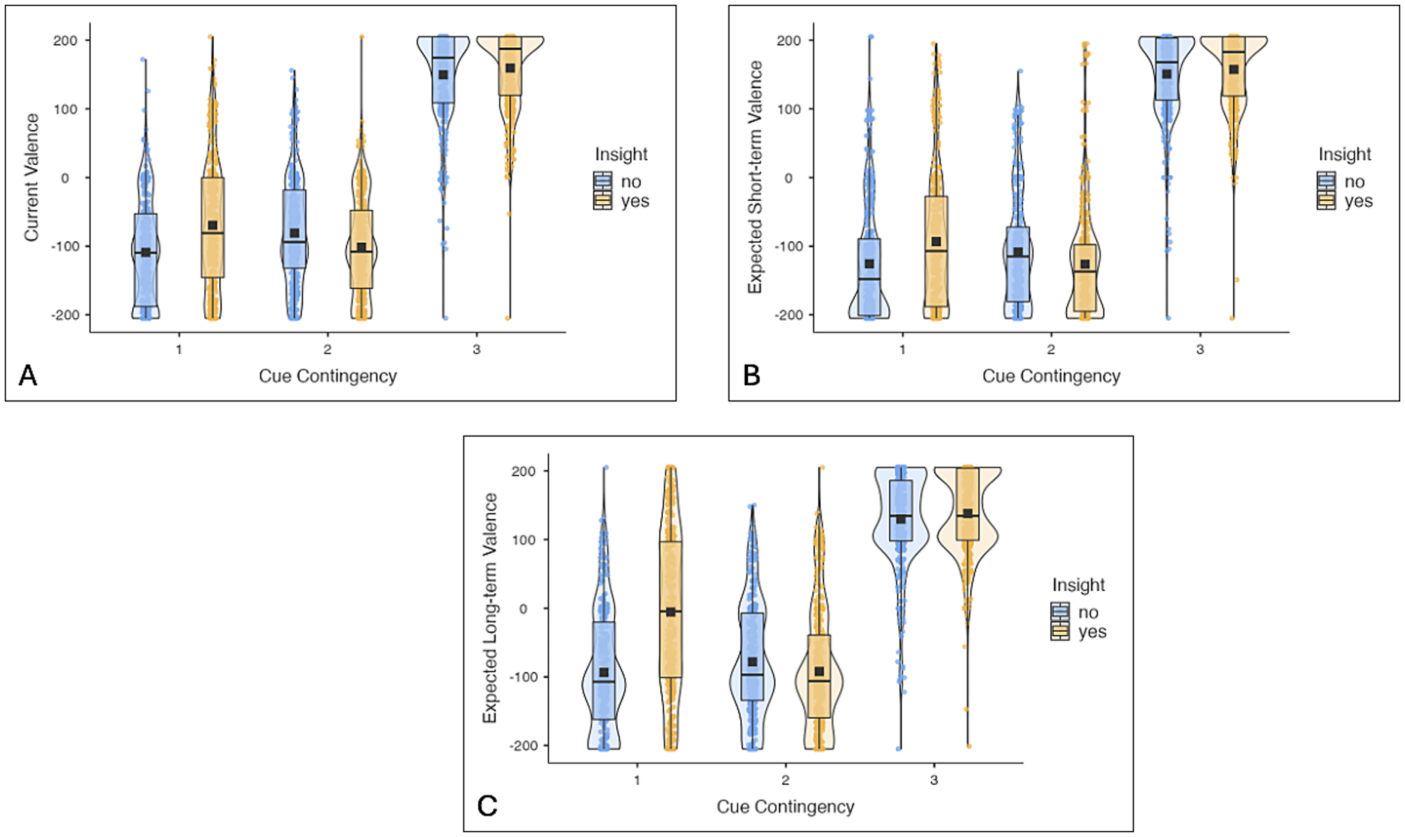
Experiment 1: Boxplots and violinplots of current **(A)**, expected short-term **(B)** and expected long-term valence ratings **(C)** depending on the cue contingency of the symbol (CC1, CC2, CC3) and whether participants had insight into the task structure. Black squares represent the mean, the black line in the box the median. More details and statistical tests can be found in the Supplementary materials.

Figure 2 presents descriptives of current, expected short-term and expected long-term valence ratings dependent on the cue contingency of the symbol and whether participants had insight into the task structure. More details and statistical tests can be found in the Supplementary materials.

##### Behavioral adaption in the questionnaire blocks

More details and statistical tests can be found in the Supplementary materials.

#### Choice prediction

**Figure 3:**
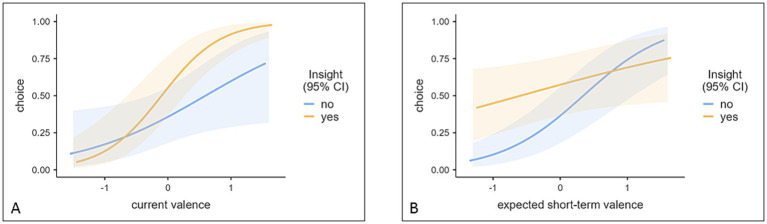
Choice prediction based on the current valence x insight interaction **(A)** and the short-term valence × insight interaction **(B)**. Transparent areas indicate 95% Confidence Intervals. Current valence and expected short-term valence are standardized to avoid large Eigenvalues.

We computed a generalized mixed effect model with participants’ choice as binary dependent variable. The link function was logit and the distribution binomial. We included the main effects of CURRENT VALENCE, SHORT-TERM-EXPECTED VALENCE, LONG-TERM-EXPECTED-VALENCE, INSIGHT and the interactions of INSIGHT with all three variables. This resulted in the formula: CHOICE ~ CURRENT VALENCE + SHORT-TERM-EXPECTED-VALENCE + LONG-TERM-EXPECTED-VALENCE +INSIGHT + INSIGHT: CURRENT_VALENCE + SHORT-TERM-EXPECTED-VALENCE: INSIGHT + LONG-TERM-EXPECTED-VALENCE: INSIGHT + (1 + LONG-TERM-EXPECTED-VALENCE + CURRENT VALENCE | SUBJECT_ID). The model was based on 2,520 observations, *R^2^_marginal_* = 0.531, *R^2^_conditional_* = 0.915, For fixed and random effect estimates see Table 1. For visualization of interactions see Figure 3.

**Table 1:** Generalized linear mixed effect estimates of the choice prediction model of Experiment 1.

Predictors	Response		
	Exp(B)	CI	*p*
(Intercept)	0.529	0.221–1.269	0.154
Long-term expected valence	7.680	2.738–21.542	**< 0.001**
Short-term expected valence	5.030	2.598–9.739	**< 0.001**
Current Valence	2.678	1.040–6.894	**0.041**
Insight	2.610	1.816–3.751	**<0.001**
Insight × Current valence	3.231	1.477–7.071	**0.003**
Insight × Short-term expected valence	0.329	0.150–0.723	**0.006**
Insight × Long-term expected valence	1.160	0.576–2.333	0.678
Random components			
Groups		Variance	ICC
Subject ID	(Intercept)	5.26	0.615
Long-term expected valence		4.80	
Current valence		3.42	
Residuals		1.00	

Fixed Effects: Estimates, Confidence Intervals (CI), and *p*-values. Random Effects: Variance Estimates for the random intercepts of Subjects and random slopes for Long-term Expected Valence and Current Valance; ICC = proportion of variance explained by between-person differences; significant results are printed in bold.

We conducted a *post hoc* power analysis to evaluate the reliability of our findings and ensure adequate sensitivity to detect significant effects in our dataset. Using a bootstrap resampling procedure, we refitted a generalized linear mixed-effects model to 1,000 resampled datasets. For each iteration, we recorded whether each predictor’s *p*-value was below 0.05. *Post hoc* power was estimated as the proportion of iterations in which each predictor was statistically significant. The analysis demonstrated high power (≥ 0.75) for most predictors, including EXPECTED LONG-TERM VALENCE (1.000), EXPECTED SHORT-TERM VALENCE (1.000), INSIGHT (1.000), CURRENT VALENCE: INSIGHT (0.826), and EXPECTED SHORT-TERM VALENCE: INSIGHT (0.758). Moderate power was observed for CURRENT VALENCE (0.539). However, power was low for the INTERCEPT (0.014) and the interaction between EXPECTED LONG-TERM VALENCE and INSIGHT (0.064). These results suggest that while the main effects and most interactions were reliably detected, certain effects, particularly the interaction involving EXPECTED LONG-TERM VALENCE and INSIGHT, may require larger sample sizes or stronger underlying effects to achieve sufficient sensitivity. This underscores the importance of interpreting these effects with caution.

Furthermore, to better understand how low statistical power affected the null effects, we analyzed binary choice data using Bayesian generalized linear mixed-effects models (GLMMs) with a Bernoulli likelihood, as implemented in the R package brms (Bürkner, 2017). All models included subject-specific random intercepts and random slopes for selected predictors. Weakly informative priors [normal(0, 1)] were placed on all fixed effects. Model estimation was performed via Markov Chain Monte Carlo sampling using Stan (Carpenter et al., 2017), with four chains, 4,000 iterations per chain, and default diagnostics checked for convergence. To evaluate the contribution of specific predictors and interactions, we conducted Bayes factor model comparisons based on marginal likelihood estimation (Kass and Raftery, 1995).

Hence, we compared a full model including the interaction between INSIGHT and EXPECTED LONG-TERM VALENCE against a reduced model without this interaction. The Bayes factor favored the null model (BF₁₀ = 0.48), providing anecdotal evidence that the interaction term does not improve model fit, and supporting the interpretation of a negligible effect.

### Discussion

In Experiment 1 we wanted to show that short-term and long-term emotional expectations independently predict choice in a recurrent gambling task. Furthermore, we wanted to show that situational demands have an impact on the predictive power of different emotional choice predictors. Therefore, we designed Experiment 1 to examine short-term and long-term expectations by manipulating presentation probabilities of one symbol depending on choices participants made. Thus, participants had to accept a small loss of points in order to maximize their outcome over the whole course of the experiment. This means, negative short-term expectations and positive long-term expectations were attached to one symbol. Participants did the same gabling task twice; however, at first they had no insight into the previously mentioned task structure. Only in the second round, they got insight into the task structure.

Results show that our experimental manipulation was successful as participants adapted their choices in the second round and, therefore, presentation probabilities changed as intended. Moreover, self-rated valence expectations changed as intended. Most importantly, long-term valence expectations were rated more positively after they had insight into the task structure. Last, participants adapted their choices in the questionnaire blocks. After they had insight into the task structure, they accepted short-term losses for the manipulated symbol more often to maximize their overall outcomes. Regarding our main research questions, results indicate that long-term, short-term and current valence are predictors of choice, which is in line with our hypotheses. Additionally, insight into the task structure decreased the predictive power of short-term valence expectations. Contrary to our hypotheses, insight into the task structure did not increase the predictive power of long-term valence but increased the predictive power of current valence.

Taken together, in Experiment 1 we could show that expected long-term valence is an additional predictor that should be considered in recurrent decision contexts. Furthermore, insight into the task structure additionally changed the predictive power of the investigated predictors. Participants still expected a negative emotional short-term outcome, but they did no longer base their choice on short-term expectations. However, after receiving insight into the task structure participants relied more on their current feelings than before. One reason could be that the information they received about the task was counterintuitive which triggered uncertainty. In other words, although participants were told the optimal gambling strategy, they still questioned the usefulness of this information as it was opposed to their intuition. This resulted in a cognitive dissonance, which makes automatic processes like current feelings more accessible. In previous experiments a similar pattern emerged after instructions were changed (see Jäger et al., 2022).

## Experiment 2

In the previous experiment, we examined short- and long-term expectations. However, participants had to accept a loss of points to win more points over the whole course of the experiment. People are loss averse over different domains and different contexts (Nabi et al., 2020) and framing effects seem to impact emotional reactions (Nygren, 1998). Having to accept a short-term loss, could potentially hamper the predictive power of long-term expectations, as losses loom larger than gains. Furthermore, participants might have stronger emotional reactions to short-term losses, which leads to a bigger predictive value of current feelings and short-term expectations. In addition to that, the reference point seems to determine whether gain seeking or loss aversion is more dominant (Mellers et al., 2021). A positive reference point encourages loss aversion whereas a negative reference point promotes gain seeking. Hence, results might not be applicable to situations in which participants have to omit positive short-term consequences to achieve long-term gains, as it could be easier for participants to omit points than to lose points for a long-term gain. Additionally, framing effects and reference points could influence the interplay of the different emotion variables.

As mentioned before, we theorize that the predictive power of the different emotion variables depends on the situational demands. We designed Experiment 1 mainly to show that long-term valence expectations are an independent predictor of choices participants make. Therefore, it is necessary to show that this effect remains stable in a point-omission-context and cannot merely be attributed to loss aversion, reference points or framing effects. To rule this out, we designed Experiment 2. We examined short-term and long-term expectations in our design. However, instead of accepting a loss of points to maximize points as in Experiment 1, participants had to omit points to do so. Participants still had to win as many points as possible and performed two rounds of the task. In the first round, they had no insight into the task structure. Only in the second round, participants were informed about short-term and long-term outcomes of their decision strategies. At the end of each round, we assessed current feelings, short-term expectations and long-term expectations prior to the respective choice in a short questionnaire block. We wanted to show four things in Experiment 2:

1. Long-term expected valence is an additional predictor of human choice besides current valence and short-term expected valence in a point-omission-context. Hence, we expected main effects of long-term expected valence, short-term expected valence and current valence.
2. Insight into the task structure does not influence the predictive power of long-term expected valence (see Experiment 1). Thus, we did not expect an interaction between insight (first vs. second round) and long-term expected valence.
3. Insight into the task structure decreases the predictive power of short-term expected valence, because the instruction defines the short-term losses as no obstacle towards the overall goal. Thus, we expected an interaction between insight (first vs. second round) and short-term expected valence.
4. Insight into the task structure should influence the predictive power of current valence as it did in Experiment 1. Hence, we expected a significant interaction between insight (first vs. second round) and current valence.

### Method

#### Participants

Sample size was determined in accordance to the sample size in Experiment 1. We wanted the effect sizes to be comparable between the two experiments, so powering Experiment 2 based on the effect size from Experiment 1 would distort this comparison. If we based Experiment 2’s sample size on Experiment 1’s effect size, we risked making one experiment over- or under-powered. This would result in significant effects that are not truly comparable across the two contexts. Hence, we decided to use the same sample size. The sample consisted of a convenience sample of 36 individuals. Participants could receive course credit for taking part in the study. Additionally, as an incentive, a raffle of two cinema vouchers for five people each was offered. The age range of the participants was from 18 to 30 years (M_age_ = 21.0; SD = 3.12), including 27 women, 8 men, and 1 non-binary person; 32 were right-handed. All participants were psychology students. All of them gave their written informed consent and were debriefed afterwards. The local ethics commission approved the study protocol.

#### Materials

The experiment consisted of two parts, which each had a Learning Phase and Predecisional Valence Questionnaire blocks, as in Experiment 1.

##### Gambling task

We employed the same gambling task but changed Cue Contingencies (CC). At the beginning of the experiment, the symbols are randomly associated with a CC that determines the winning probabilities of the symbols. Symbols with CC 2 lead to negative results 90% of the time (i.e., −15 points). Symbols with CC1 and CC 3, on the other hand, are positive 90% of the time (i.e., +15 points). Moreover, we changed the urn model: 10 of balls are labeled with CCs 2 and 3 each. At the same time, 15 balls are assigned to CC 1. While the number of balls labeled with CCs 2 and 3 change, the number of balls with CC1 remain the same: If a participant decides to play the CC1 symbol (90% positive outcome), one CC3 symbol (90% positive outcome) is removed from the urn and one CC2 symbol (90% negative outcome) is added to the urn. This reduces the probability of occurrence of CC3 symbols. Conversely, if they decide not to play the CC1 symbol, it is the other way round. One CC3 symbol is added to the urn while a CC2 symbol is removed. This increases the probability of occurrence of CC3 symbols. Consequently, the probability of occurrence of the CC2 symbol increases whenever the positive consequence of +15 points is accepted. After several trials, the participants find themselves in a situation where they constantly must avoid CC2 symbols. This means, over the whole course of the experiment it is beneficial to omit the wins of the CC1 symbols to get more opportunities to win points with the other symbols.

##### Predecisional valence questionnaire task

We did not change the task except for the cue contingency structure in comparison to Experiment 1.

#### Procedure

The procedure remained the same as in Experiment 1. We only adjusted instructions according to the new cue contingency structure.

##### Analysis plan

We did not change our analysis strategy. See Experiment 1 for more details.

### Results

#### Manipulation check

##### Presentation probabilities in the learning blocks for CC2 and CC3 symbols

**Figure 4:**
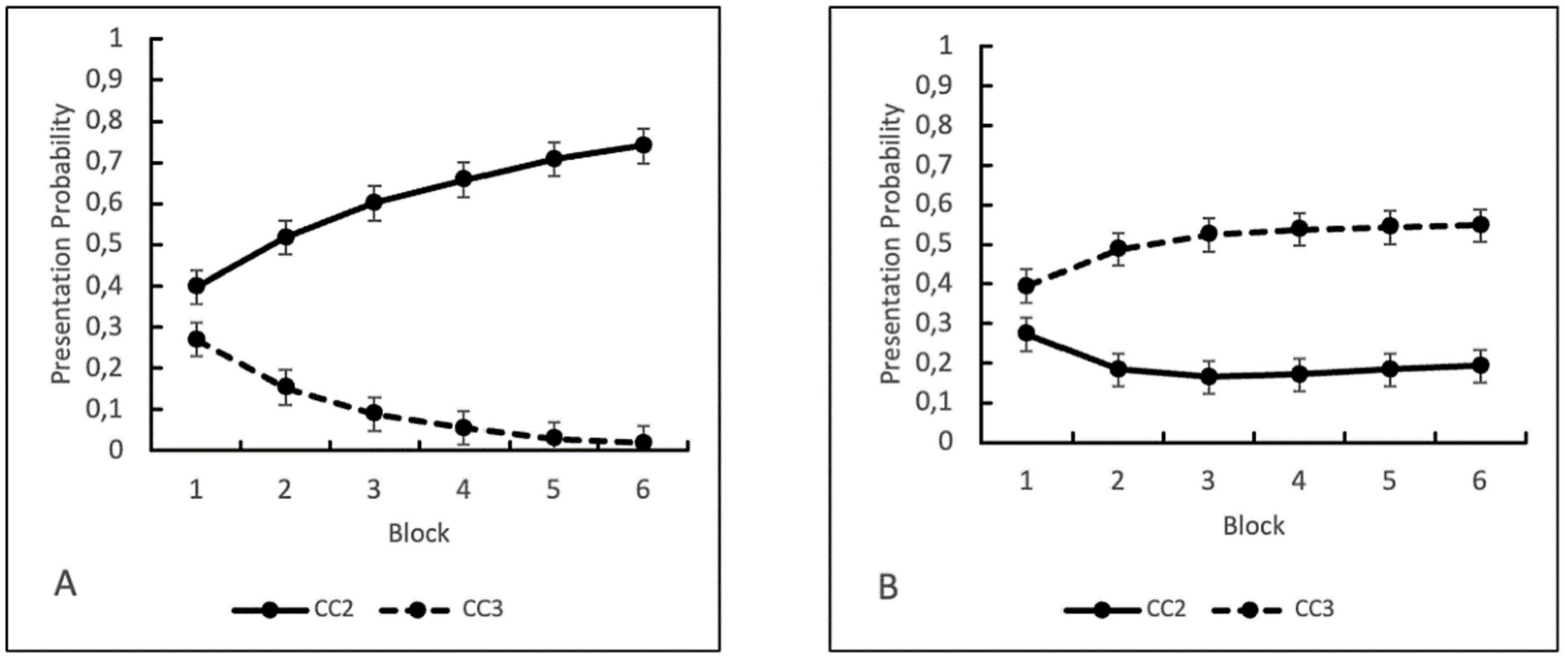
Presentation Probabilities in Experiment 2 for the CC2 and CC3 symbols depending on Block and Round of the gambling task. **(A)** Round 1 before insight; **(B)** Round 2 after insight. Error-bars indicate Confidence Intervals.

We computed a linear mixed effects model for the presentation probabilities of the CC2 and CC3 symbols. Presentation probabilities for each trial were determined using the same formulas as in Experiment 1. We included the interaction of BLOCKxROUNDxSYMBOL, BLOCKxSYMBOL, ROUNDxSYMBOL, BLOCKxROUND and the main effects of SYMBOL, BLOCK and ROUND as fixed effects in our model. The model converged including SUBJECT_ID as a random effect, which resulted in the formula of PRESENTATION PROBABILITY ~ BLOCK*ROUND*SYMBOL + (1|SUBJECT_ID). Estimates were fit by REML. There was a significant disordinal BLOCK*ROUND*SYMBOL interaction, *F*(5, 23,269) = 850,5, *p* < 0.001 (see Figure 4). The interaction revealed that in Round 1, before participants had insight into the task structure, the mean presentation probabilities of CC2 symbols increased over the blocks, *M_Block1_* = 0.0.397, *CI* = [0.387, 0.408], *M_Block2_* = 0.517, *CI* = [0.506, 0.527], *M_Block3_* = 0.6011, *CI* = [0.591, 0.612], *M_Block4_* = 0.658, *CI* = [0.647, 0.668], *M_Block5_* = 0.707, *CI* = [0.697, 0.718], *M_Block6_* = 0.740, *CI* = [0.730, 0.751]. The mean presentation probabilities of CC3 symbols decreased and then remained stable over the blocks, *M_Block1_* = 0.270, *CI* = [0.259, 0.280], *M_Block2_* = 0.1533, *CI* = [0.143, 0.164], *M_Block3_* = 0.090, *CI* = [0.079, 0.100], *M_Block4_* = 0.055, *CI* = [0.044, 0.066], *M_Block5_* = 0.028, *CI* = [0.018, 0.039], *M_Block6_* = 0.019, *CI* = [0.008, 0.029]. In contrast in Round 2, after participants had had insight into the task structure, the mean presentation probabilities of CC2 symbols remained stable after an initial decrease, *M_Block1_* = 0.272, *CI* = [0.262, 0.283], *M_Block2_* = 0.184, *CI* = [0.173, 0.194], *M_Block3_* = 0.164, *CI* = [0.154, 0.175], *M_Block4_* = 0.171, *CI* = [0.160, 0.182], *M_Block5_* = 0.1833, *CI* = [0.173, 0.194], *M_Block6_* = 0.193, *CI* = [0.182, 0.203]. The mean presentation probabilities of CC3 symbols remained stable after an initial increase, *M_Block1_* = 0.394, *CI* = [0.384, 0.405], *M_Block2_* = 0.487, *CI* = [0.476, 0.498], *M_Block3_* = 0.524, *CI* = [0.514, 0.535], *M_Block4_* = 0.538, *CI* = [0.527, 0.548], *M_Block5_* = 0.543, *CI* = [0.533, 0.5536], *M_Block6_* = 0.548, *CI* = [0.537, 0.558]. See also Figure 4.

##### Valence ratings

**Figure 5:**
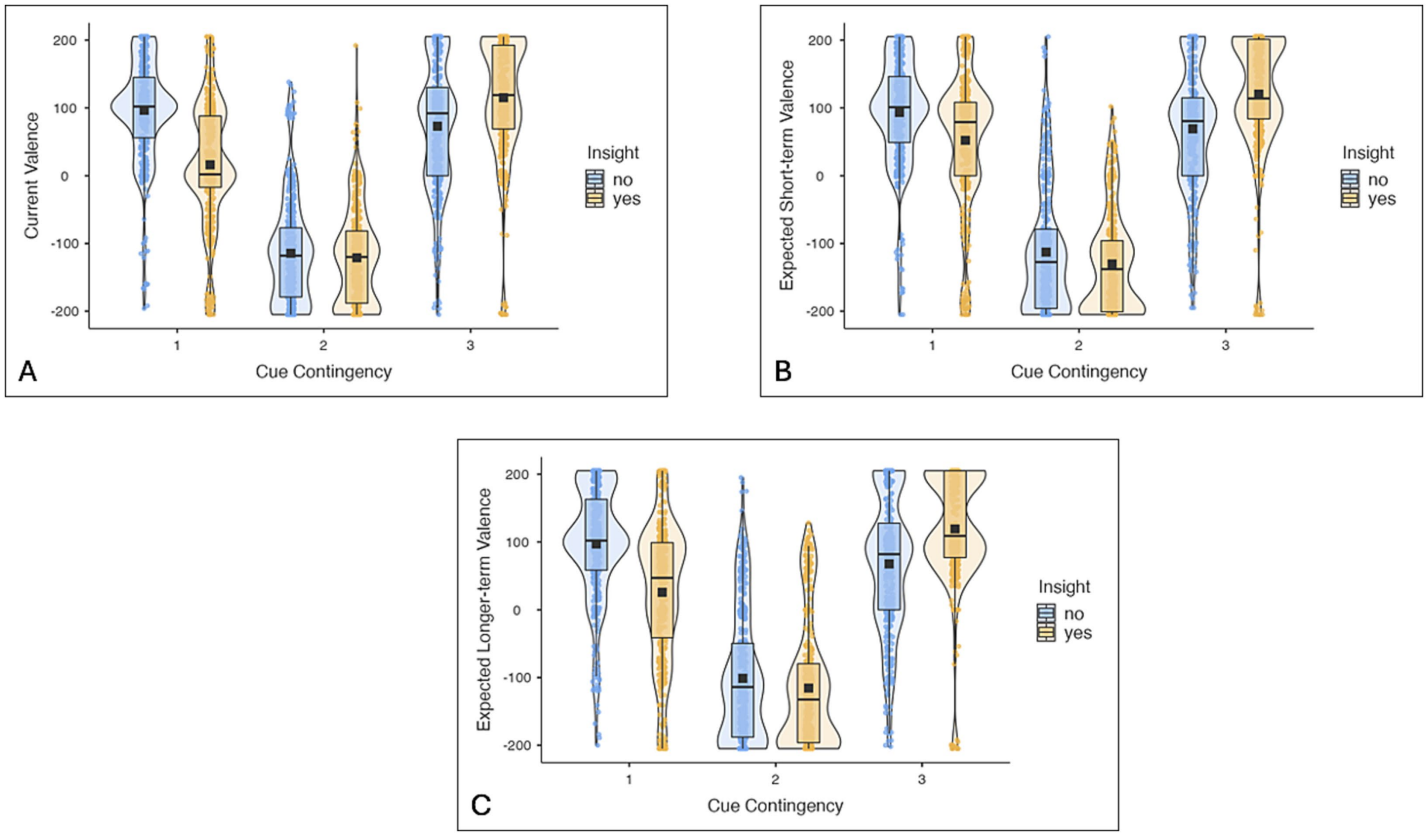
Experiment 2: Boxplots and violinplots of current **(A)**, expected short-term **(B)** and expected long-term valence ratings **(C)** depending on the cue contingency of the symbol (CC1, CC2, CC3) and whether participants had insight into the task structure. Black squares represent the mean, the black line in the box the median. More details and statistical tests can be found in the Supplementary materials.

Figure 5 presents descriptives of current, expected short-term and expected long-term valence ratings depending on the cue contingency of the symbol and whether participants had insight into the task structure. More details and statistical tests can be found in the Supplementary materials.

##### Behavioral adaption in the questionnaire blocks

More details and statistical tests can be found in the Supplementary materials.

#### Choice prediction

**Figure 6:**
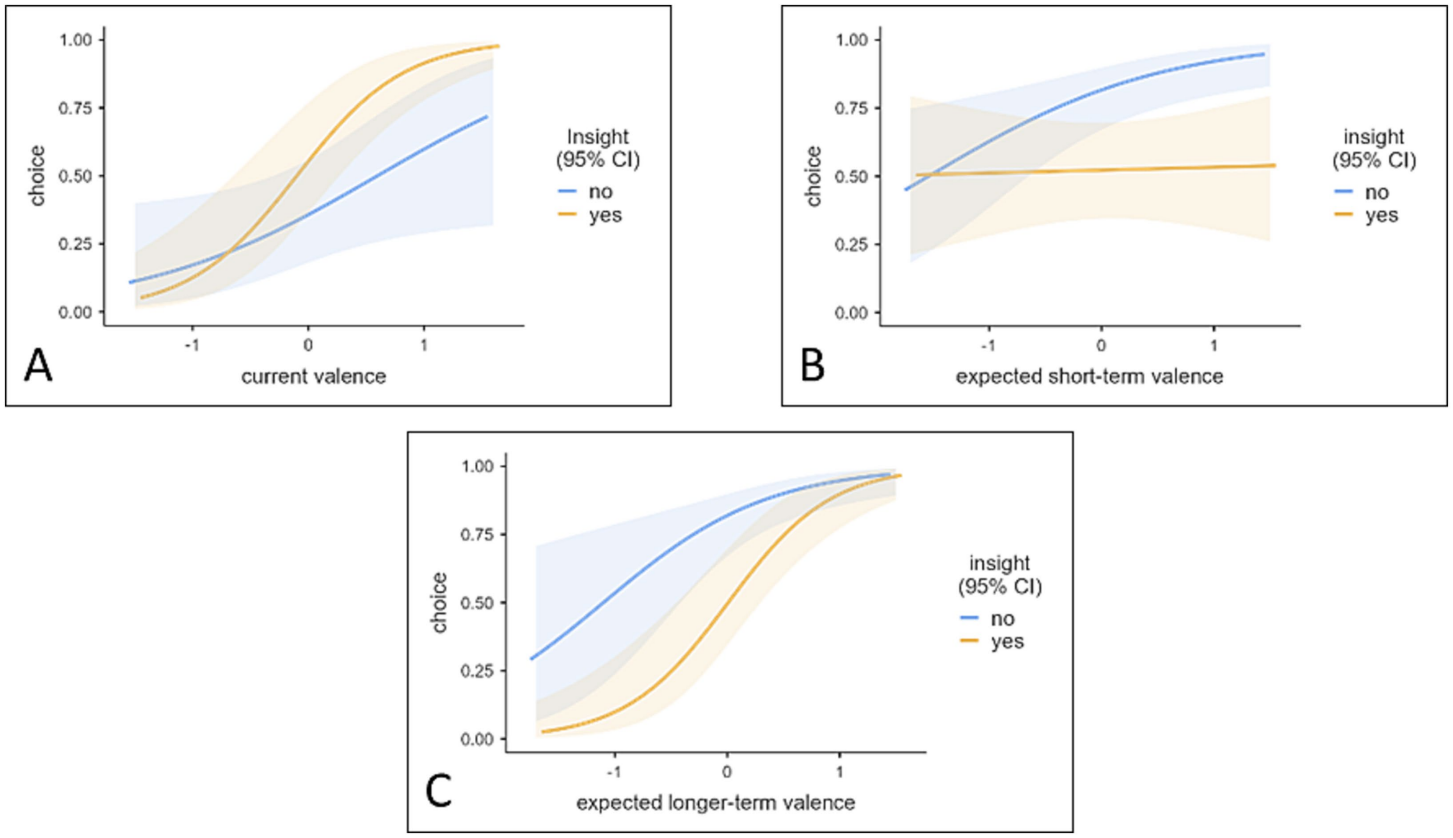
Choice prediction based on the current valence x insight interaction **(A)**, the short-term expected valence x insight interaction **(B)**, and the long-term expected valence x insight interaction **(C)**. Transparent areas indicate 95% Confidence Intervals. Current valence, expected short-term valence, expected long-term valence are standardized to avoid large Eigenvalues.

We computed a generalized mixed effect model with participants’ choice as binary dependent variable. The link function was logit and the distribution binomial. We included the main effects of CURRENT VALENCE, SHORT-TERM-EXPECTED VALENCE, LONG-TERM-EXPECTED-VALENCE, INSIGHT and the interactions with INSIGHT with all three variables. This resulted in the formula: CHOICE ~ CURRENT VALENCE + SHORT-TERM-EXPECTED-VALENCE + LONG-TERM-EXPECTED-VALENCE +INSIGHT + INSIGHT: CURRENT VALENCE + SHORT-TERM-EXPECTED-VALENCE: INSIGHT + LONG-TERM-EXPECTED-VALENCE: INSIGHT + (1 + LONG-TERM-EXPECTED-VALENCE + CURRENT VALENCE +SHORT-TERM-EXPECTED_VALENCE | SUBJECT_ID). The model was based on 23,592 observations, *R^2^_marginal_* = 0.405, *R^2^_conditional_* = 0.897. For fixed and random effect estimates see Table 2. Significant interactions are displayed in Figure 6.

**Table 2:** Generalized linear mixed effect estimates of the choice prediction model of Experiment 2.

Predictors	Response		
	Exp(B)	CI	*p*
(Intercept)	2.159	1.054–4.423	**0.035**
Long-term expected valence	5.955	2.609–13.593	**< 0.001**
Short-term expected valence	1.660	0.960–2.869	0.069
Current valence	3.840	1.704–8.656	**0.001**
Insight	0.258	0.186–0.359	**<0.001**
Insight × Current valence	2.791	1.544–5.046	**<0.001**
Insight × Short-term expected valence	0.395	0.190–0.821	**0.013**
Insight × Long-term expected valence	2.273	1.158–4.462	**0.017**
Random components			
Groups		Variance	ICC
Subject ID	(Intercept)	4.29	0.566
Long-term expected valence		3.84	
Current valence		3.63	
Short-term expected valence		0.63	
Residuals		1.00	

Fixed Effects: Estimates, Confidence Intervals (CI), and *p*-values. Random Effects: Variance Estimates for the random intercepts of subjects and random slopes for Long-term Expected Valence, Current Valance, and Short-Term Expected Valence; ICC = proportion of variance explained by between-person differences; significant results are printed in bold.

To assess the sensitivity and robustness of our analysis in the second experiment, we conducted a *post hoc* power analysis using a bootstrap resampling procedure. The generalized linear mixed-effects model was refitted to 1,000 resampled datasets, and the significance of each predictor was evaluated in each iteration. Power was estimated as the proportion of iterations in which each predictor reached statistical significance (*p* < 0.05). The results showed high power for CURRENT VALENCE (0.96), EXPECTED LONG-TERM VALENCE (1.00), and INSIGHT (1.00), as well as for the interaction between CURRENT VALENCE and INSIGHT (0.85). Moderate power was observed for the interactions of EXPECTED SHORT-TERM VALENCE with INSIGHT (0.56) and EXPECTED LONG-TERM VALENCE with INSIGHT (0.59). In contrast, power was low for EXPECTED SHORT-TERM VALENCE (0.08) and the INTERCEPT (0.33). These findings suggest the model had sufficient sensitivity to detect main effects and key interactions but limited power for certain predictors, particularly for EXPECTED SHORT-TERM VALENCE and its interaction with INSIGHT.

We applied the same Bayesian model comparison approach as in Experiment 1 to assess the contribution of EXPECTED SHORT-TERM VALENCE in Experiment 2. Specifically, we compared a full model to a reduced model that excluded only the main effect of EXPECTED SHORT-TERM VALENCE. The resulting Bayes factor (BF₁₀ = 0.32) indicates moderate evidence in favor of the null model, suggesting that EXPECTED SHORT-TERM VALENCE does not meaningfully contribute to explaining choice behavior in this experiment.

### Discussion

In Experiment 2, we wanted to replicate the findings of Experiment 1 in a point-omission context. Hence, we designed Experiment 2 to examine short-term and long-term expectations by manipulating presentation probabilities of the symbols depending on choices participants made. Thus, participants had to omit points to maximize their outcome over the whole course of the experiment. In contrast to Experiment 1, positive short-term expectations and negative long-term expectations were attached to the same symbol. Participants performed the same gabling task twice; however, at first, they had no insight into the previously mentioned task structure. Only in the second round, they were given insight into the task structure.

In short, the manipulation worked as planned. Participants adapted their choices in the second round to the instruction. Moreover, self-rated valence expectations changed as expected. Most importantly, long-term valence expectations for the manipulated symbol were rated more positively after participants gained insight into the task structure. Last, participants adapted their choices in the questionnaire blocks. After they had had insight into the task structure, they omitted points for the manipulated symbol more often to maximize their overall outcomes. All in all, results remained stable: We found main effects for current and long-term expected valence, and interaction effects for current valence and short-term expected valence with insight into the task structure. Nevertheless, there were also some findings contrary to our hypotheses. We did not find a main effect of expected valence on gambling choice and long-term expected valence showed an interaction effect with insight into the task-structure in a point omission context.

One reason that we did not find a main effect for expected valence could be loss aversion. It relates to expected emotions and is present in contexts of immediate experience of realized gains and losses (Sokol-Hessner and Rutledge, 2019). Hence, loss aversion might have been more prominent in the first experiment, which lead to a stronger reliance on short-term expected valence, even after participants got insight into the task structure in Experiment 1. However, in Experiment 2, short-term expectations did no longer influence choices participants made after participants got insight into the task structure. In Experiment 1 the influence of expected valence diminished but was still present despite insight into the task structure, which might mean that it was easier for participants to omit points than to accept a loss of points. The framing of losing points seems to trigger a stronger reliance on short-term expectations, while the framing of omitting points for a long-term goal seems to eradicate the reliance on short-term expectations. This further adds to the framing effect literature (see also Nabi et al., 2020).

Moreover, we found an interaction effect for long-term expected valence and insight as we originally expected in the hypotheses of Experiment 1. Again, loss aversion is a reasonable explanation for this discrepancy. In both experiments, we found a main effect for long-term expected valence. In Experiment 2, the influence on choice of long-term expected valence even became stronger after participants got insight into the task structure. However, in Experiment 1, a similar effect could not be observed, as participants had to accept losses. This might have hampered a stronger reliance on long-term expectations. We will discuss this in further detail in the general discussion section.

## General discussion

In two experiments, we could show that the human subjective feeling system is capable of adapting to different choice situations. Current valence, short-term valence expectations and long-term valence expectations were significant predictors in different choice contexts. Our findings clearly show that the predictive value of each emotional variable depends on the choice situation. Looking at the random effects of our models shows that the predictive power of each construct is highly dependent on individual differences between participants. Furthermore, changing the choice situation by providing information on the task structure changes the predictive value of the valence constructs depending on the context (losing points, Experiment 1 vs. omitting points, Experiment 2). After getting insight into the task structure, losing points opposed to omitting points seems to make a reliance on short-term expectations more likely. Omitting points increases the reliance on long-term expectations after insight into the task structure. For both contexts, the predictive power of current valence increased after participants got insight. One reason could be that the instruction proposed a counterintuitive, optimal gambling strategy which generated uncertainty. Although participants knew the optimal gambling strategy, it was discordant with their intuition. This resulted in a cognitive dissonance, which makes automatic processes like current feelings more accessible. In previous experiments a similar pattern emerged after instructions were changed (see Jäger et al., 2022).

Overall, our findings align with prior research on the influence of emotional valence on risky decision-making, while expanding on previous work by incorporating both current and expected emotional states. Charpentier et al. (2016) demonstrated that expected valence—how positively participants anticipated feeling after the decision—was a strong predictor of gambling behavior, with higher expectations of positive emotion leading to increased gambling. Mellers et al. (1997) found similar results, reinforcing the role of expected emotions, though neither study investigated the role of current valence (emotions felt at the moment of decision-making). Building on this, Schlösser et al. (2013) examined the role of immediate emotions, in contrast to anticipated emotions. Their findings support the risk-as-feelings hypothesis, which argues that many risky decisions are influenced not only by anticipated emotions but also by the “hot” visceral feelings individuals experience in the moment of decision-making. Schlösser et al. (2013) found that these immediate emotions predicted decisions beyond anticipated emotions or subjective probabilities. In other words, decisions were driven by how participants felt about the decision options themselves, rather than solely by their predictions of future emotional outcomes or the perceived probabilities of those outcomes. This emphasizes the powerful influence of current emotional states on risky choices, a finding that resonates with our results, which also show that both current and expected valence significantly predict gambling behavior. Jäger et al. (2022) further supported this by showing that current and expected valence interact to predict choices, underscoring the dynamic relationship between current feelings and future emotional expectations in decision-making. Our findings add to the literature in showing that expected emotional states can be differentiated in those occurring immediately after a recurrent decision is made and in long-term expected emotional states. We could also demonstrate that both time perspectives differ regarding their influence on decision making, and that their effect depends on the structure of the decision task.

To expand on this, our results resonate with Seth (2013) model of interoceptive inference, which proposes that emotions arise from the brain’s predictive modeling of internal bodily states. According to this framework, subjective feeling states (such as current valence) reflect the brain’s top-down predictions about interoceptive input, which are updated through prediction errors. This predictive model suggests that uncertainty or dissonance—such as the tension between intuitive and instructed strategies in our task—may enhance the salience of interoceptive signals, thereby increasing the impact of current feelings on decision-making.

Our findings are in line with the work by Schneider et al. (2016), who showed that past emotional experiences, such as a series of losses or gains, affect future risk-taking behavior. Their work suggests that emotional outcomes shift reference points, influencing future decisions—a concept that ties into how current and expected valence can shape gambling choices in our study. Prietzel (2020) reviewed the impact of emotions on decision-making through the lens of Prospect Theory, finding that positively valenced emotions generally lead to increased risk-taking in the gain domain. Prietzel (2020) also emphasized the complexity of negatively valenced emotions, which exhibit varied effects depending on the context. The review emphasized the distinction between integral emotions (emotions directly tied to the decision) and incidental emotions (unrelated to the decision). Integral emotions tend to lead to decisions that deviate more from the predictions of Prospect Theory, with greater independence from scope and probability. Hence, our findings suggest that the subjective valuation system is highly adaptable to various choice situations. The interplay between current and expected valence in predicting decisions highlights the flexibility of emotional influences on risky decision-making. Future research should focus on identifying the conditions under which different emotional states interact and how task structures and contexts shape these dynamics. These factors hold great potential for advancing our understanding of emotion-driven choices.

In light of our findings, it is also important to consider the role of language and internal bodily states in shaping emotional experiences and their impact on decision-making. Brooks et al. (2017) demonstrated that the presence of emotion words can modulate neural activity during emotional processing by enhancing the activation of semantic brain regions (e.g., inferior frontal and temporal cortices) and reducing amygdala responses. This suggests that the conceptualization of emotion through language—such as labeling a symbol as “good” or “bad”—may influence how emotional information is accessed and used in decision-making. In our study, participants were given structured verbal instructions that framed the task in emotionally salient ways (e.g., “losing” vs. “omitting” points), which likely affected how they conceptualized and subsequently integrated their emotional experiences. The increased influence of current valence after instruction could thus reflect a language-mediated process in which emotional states became more accessible and actionable through verbal categorization.

One limitation of our study is that, while we conducted a power analysis, it is possible that our experiments were underpowered to detect smaller effects. Nevertheless, our post-hoc power analysis reached sufficient power for most effects. Significant effects with a post-hoc power below 0.75 should be interpreted with caution. It is also possible that our self-report measures captured verbal evaluations of the reward contingencies rather than participants’ true subjective feelings. While self-report offers the advantage of directly accessing participants’ conscious reflections and is relatively easy to administer, it is prone to biases such as demand characteristics and may not fully capture unconscious or more automatic emotional responses. In contrast, physiological measures or behavioral proxies could offer more objective insight into emotional processing but often lack the specificity of self-report when it comes to understanding how participants consciously interpret their emotional states. Despite these limitations, we believe that self-report still provides meaningful data. Relatedly, we did not assess participants’ arousal levels, focusing exclusively on valence judgments. This decision was based on findings from previous work (Jäger et al., 2020, 2022) and pilot testing, which indicated that self-reported arousal did not systematically influence participants’ choices. To reduce task complexity and participant burden, we chose to omit arousal ratings. However, we acknowledge that arousal may play a relevant role in other contexts or populations, and future studies should consider its inclusion to provide a more comprehensive picture of emotional processing. Lastly, we were unable to fit the maximal random-effects structure in our regression models, which may increase the risk of anticonservative results (Barr et al., 2013). While we included relevant random slopes where possible, future research should aim for a more comprehensive random-effects structure to minimize the likelihood of Type 1 errors. Additionally, one potential limitation concerns the influence of handedness on valence judgments. Prior research (Casasanto, 2009; Milhau et al., 2013, 2015) has shown that individuals often associate positive concepts with their dominant side, suggesting that handedness can systematically shape emotional-laterality associations. While we did not explicitly assess handedness, we accounted for individual variability—such as motor fluency or dominant hand—by including participant identity as a random effect in our models. This statistical approach captures stable individual response patterns, potentially including those related to handedness. Nonetheless, the absence of a direct handedness measure means we cannot disentangle its specific contribution. Future research could benefit from incorporating handedness as an explicit variable to examine its role more precisely. Finally, the generalizability of our findings is limited by the use of a laboratory-based gambling task and a homogeneous student sample. While this allowed for tight experimental control, it may not fully reflect the complexity of real-world decision-making or emotional processes in more diverse populations. Future research should test whether our results replicate in ecologically valid contexts and with more heterogeneous samples.

In conclusion, our study demonstrates that the subjective feeling system adapts to different choice situations, with current valence, short-term, and long-term valence expectations significantly predicting risky decisions. The predictive power of these emotional variables is context-dependent, influenced by task structure and individual differences. Our findings highlight the flexibility of emotional influences in decision-making and emphasize the importance of understanding how various contexts shape the interaction between current and expected emotions. Future research should continue to explore these dynamics to further deepen our understanding of emotion-driven choices.

## Data Availability

The datasets presented in this study can be found in online repositories. The names of the repository/repositories and accession number(s) can be found at: https://osf.io/ypc7v/.
