# Supplementary material for: Time matters: on the predictive power of current, short- and long-term expected valence in an experience based learning task
Source: Front Psychol. 2025 Sep 30;16:1570369. doi: 10.3389/fpsyg.2025.1570369 (PMC12520190; doi:10.3389/fpsyg.2025.1570369)
Supplement: Supplementary file 1 [file Table_1.DOCX]

**Supplementary Materials**

1. *Results of statistical tests for valence ratings and behavioral adaption in the questionnaire blocks in Experiment 1*

*Valence Ratings.* We computed linear mixed effects models for each valence perspective (current valence, expected short-term valence, expected long-term valence). We included the interaction of INSIGHTxCUE_CONTINGENCY and the main effects of INSIGHT and CUE_CONTINGENCY as fixed effects in our models. The three models all converged including SUBJECT_ID and SYMBOL_ID as random effects, which resulted in the formula of VALENCE-PERSPECTIVE ~ INSIGHT*CUE_CONTINGENCY + (1|SUBJECT_ID) + (1|SYMBOL_ID). Estimates were fit by REML.

For *Current Valence Ratings*, the model was based on 2520 observations, *R^2^_marginal_* = 0.706, *R^2^_conditional_* = 0.735, *ICC_SUBJECT_ID_* = 0.087, *ICC_SYMBOL_ID_* = 0.014. There was a significant CUE_CONTINGENCYxINSIGHT interaction, *F*(2, 2260) = 3192.0, *p* < .001, and two significant main effects: INSIGHT, *F*(1, 2477) = 11.4, *p* < .001, and CUE_CONTINGENCY, *F*(1, 2477) = 11.4, *p* < .001. The interaction revealed that CC3 symbols, *M* = 154.1, *CI* = [141, 167.7] differed significantly from CC2 symbols, *M* = -90.2, *CI* = [-104, -76.6], *t*(2053) = -65.9, *p* < .001, and CC1 symbols, *M* = -89.0, *CI* = [-103, -75.4] *,t*(2479) = -70.2, *p* < .001, regardless of the factor INSIGHT. However, for CC2 and CC1 symbols there was a significant interaction with INSIGHT, *F*(1, 1639) = 86.2, *p* < .001. CC1 symbols had lower current valence ratings in the questionnaire block before they got insight into the task structure, *M* = -108.7, *CI* = [-122.8, -94.6] than after they had insight in the second questionnaire block, *M* = -69.3, *CI* = [-83.4, -55.2], *t*(1639) = -8.67, *p* < .001. For CC2 symbols, it was the other way around. They had higher current valence ratings in the questionnaire block before they got insight into the task structure, *M* = -79.9, *CI* = [-94.1, -65.7] than after they had insight in the second questionnaire block, *M* = -100.6, *CI* = [-114.8, -86.4], *t*(1639) = 4.52, *p* < .001.

 For *Short-Term Expected Valence Ratings*, the model was based on 2520 observations, *R^2^_marginal_* = 0.713, *R^2^_conditional_* = 0.767, *ICC_SUBJECT_ID_* = 0.168, *ICC_SYMBOL_ID_* = 0.029. There was a significant CUE_CONTINGENCYxINSIGHT interaction, *F*(2, 2477) = 25.81, *p* < .001, and two significant main effects: INSIGHT, *F*(1, 2477) = 6.16, *p* = .013, and CUE_CONTINGENCY, *F*(1, 2438) = 3675.29, *p* < .001. The interaction revealed that CC3 symbols, *M* = 152, *CI* = [132, 171.3] differed significantly from CC2 symbols, *M* = -116, *CI* = [-135, -96.1], *t*(2379) = -71.4, *p* < .001, and CC1 symbols, *M* = -111, *CI* = [-130, -91.2] *,t*(2478) = -75.0, *p* < .001, regardless of the factor INSIGHT. However, for CC2 and CC1 symbols there was a significant interaction with INSIGHT, *F*(1, 1636) = 72.1, *p* < .001. CC1 symbols had lower short-term expected valence ratings in the questionnaire block before they got insight into the task structure, *M* = -126.8, *CI* = [-147, -107.1] than after they had insight in the second questionnaire block, *M* = -94.5, *CI* = [-114, -74.7], *t*(2477) = -6.6, *p* < .001. For CC2 symbols, it was the other way around. They had higher short-term expected valence ratings in the questionnaire block before they got insight into the task structure, *M* = -106.6, *CI* = [-126, -86.8] than after they had insight in the second questionnaire block, *M* = -124.4, *CI* = [-144, -104.6], *t*(2477) = 3.6, *p* = .005.

For *Long-Term Expected Valence Ratings*, the model was based on 2520 observations, *R^2^_marginal_* = 0.567, *R^2^_conditional_* = 0.634, *ICC_SUBJECT_ID_* = 0.142, *ICC_SYMBOL_ID_* = 0.016. There was a significant CUE_CONTINGENCYxINSIGHT interaction, *F*(2, 2477) = 96.0, *p* < .001, and two significant main effects: INSIGHT, *F*(1, 2477) = 75.0, *p* < .001, and CUE_CONTINGENCY, *F*(1, 2324) = 1714.1, *p* < .001. The interaction revealed that CC3 symbols, *M* = 133.1, *CI* = [115.8, 150.4] differed significantly from CC2 symbols, *M* = -84.7, *CI* = [-102.0, -67.3], *t*(2160) = -52.4, *p* < .001, and CC1 symbols, *M* = -49.3, *CI* = [-66.6, -32.0] *, t*(2478) = -47.0, *p* < .001, regardless of the factor INSIGHT. However, the CC1 symbol had higher ratings after getting insight into the task structure, *M* = -5.3, *CI* = [-23.2, 12.6], compared to before, *M* = -93.4, *CI* = [-111.3, -75.4]*, t*(2478) = -47.0, *p* < .001. For CC2 symbols before, *M* = -77.7, *CI* = [-95.6, -59.7]*,* and after insight, *M* = -91.7, *CI* = [-109.7, -73.7]*,* measurements did not differ significantly, *t*(2477) = 2.56, *p* = .159. For CC3 symbols before, *M* = 129.0, *CI* = [111.1, 146.9]*,* and after insight, *M* = 137.2, *CI* = [119.3, 155.1]*,* measurements did not differ significantly, *t*(2477) = -1.49, *p* = 1.00.

*Behavioral Adaption in the Questionnaire Block.* We computed a generalized mixed effect model with participants’ choice as binary dependent variable. The link function was logit and the distribution binomial. We included the interaction of INSIGHTxCUE_CONTINGENCY and the main effects of INSIGHT and CUE_CONTINGENCY as fixed effects in our model. This resulted in de following formula: CHOICE ~ INSIGHT*CUE_CONTINGENCY + (1+CUE_CONTINGENCY|SUBJECT_ID). The model was based on 2520 observations, *R^2^_marginal_* = 0.537, *R^2^_conditional_* = 0.931, *ICC_SUBJECT_ID_* = 0.728. There was a significant INSIGHTxCUE_CONTINGENCY interaction, *X^2^(2, N = 35) = 148.4, p < .001,* and two significant main effects: INSIGHT, *X^2^(1, N = 35) = 36.9, p < .001,* and CUE_CONTINGENCY, *X^2^(2, N = 35) = 440.1, p < .001*. To clarify the interaction we did post-hoc comparisons, which revealed that CC3 symbols, *Prob.* = .999, *CI* = [0.988, 1.000]*,* had the highest gambling probabilities compared to CC2 symbols, *Prob.* = .052, *CI* = [0.015, 0.16]*, z* = -5.43, *p* < .001, and CC1 symbols, *Prob.* = .272, *CI* = [0.114, 0.522]*, z* = -4.61, *p* < .001. However, CC1 symbols, *Prob.* = .064, *CI* = [0.022, 0.174]*,* had equally low gambling probabilities as CC2 symbols, *Prob.* = 0.093, *CI* = [0.028, 0.265]*, z* = -0.838, *p* = 1.00, before participants had insight into the task structure. After they received insight, CC1 symbols, *Prob.* = .672, *CI* = [0.410, 0.858]*,* had significantly higher gambling probabilities than before, *Prob.* = .064, *CI* = [0.022, 0.174]*, z* = -12.49, *p* < .001, but still a lower gambling probability than CC3 symbols, *Prob.* = .999, *CI* = [0.995, 1.00]*, z* = -4.074, *p* < .001.

1. *Results of statistical tests for valence ratings and behavioral adaption in the questionnaire blocks in Experiment 2*

*Valence Ratings.* We computed linear mixed effects models for each valence perspective (current valence, expected short-term valence, expected long-term valence). We included the interaction of INSIGHTxCUE_CONTINGENCY and the main effects of INSIGHT and CUE_CONTINGENCY as fixed effects in our models. The three models all converged including SUBJECT_ID and SYMBOL_ID as random effects, which resulted in the formula of VALENCE-PERSPECTIVE ~ INSIGHT*CUE_CONTINGENCY + (1|SUBJECT_ID) + (1|SYMBOL_ID). Estimates were fit by REML.

For *Current Valence Ratings*, the model was based on 2592 observations, *R^2^_marginal_* = 0.573, *R^2^_conditional_* = 0.642, *ICC_SUBJECT_ID_* = 0.151, *ICC_SYMBOL_ID_* = 0.013. There was a significant CUE_CONTINGENCYxINSIGHT interaction, *F*(2, 2548) = 142.3, *p* < .001, and two significant main effects: INSIGHT, *F*(1, 2548) = 25.2, *p* < .001, and CUE_CONTINGENCY, *F*(1, 2457) = 1805.6, *p* < .001. The interaction revealed that CC2 symbols before participants got insight into the task structure, *M* = -114.9, *CI* = [-131.21,-98.5] did not differ significantly from CC2 symbols after participants got insight into the task structure, *M* = -121.6, *CI* = [-137.9,-105.2], *t*(2548) = 1.30, *p* = 1.00. However, CC1 symbols, before participants got insight into the task structure, *M* = 96.6, *CI* = [80.3, 112.9], had higher ratings than CC1 symbols, after participants got insight into the task structure, *M* = 16.3, *CI* = [-0.06, 32.6], *t*(2548) = 15.5, *p* < .001. For CC3 symbols it was the other way around, before participants got insight into the task structure, CC3 symbols had lower ratings , *M* = 72.7, *CI* = [56.3, 89.0], than after they gained insight, *M* = 114.8, *CI* = [98.4, 131.1], *t*(2548) = -8.15, *p* < .001.

For *Short-Term Expected Valence Ratings*, the model was based on 2592 observations, *R^2^_marginal_* = 0.559, *R^2^_conditional_* = 0.630, *ICC_SUBJECT_ID_* = 0.154, *ICC_SYMBOL_ID_* = 0.011. There was a significant CUE_CONTINGENCYxINSIGHT interaction, *F*(2, 2548) = 78.7, *p* < .001, and one significant main effects: INSIGHT, *F*(1, 2548) < 1, and CUE_CONTINGENCY, *F*(1, 2422) = 1780.7, *p* < .001. The interaction revealed that CC2 symbols before participants got insight into the task structure, *M* = -111.5, *CI* = [-128.2, -94.8] differed significantly from CC2 symbols after participants got insight into the task structure, *M* = -121.6, *CI* = [-145.7, -112.3], *t*(2548) = 3.24, *p* = .018. CC1 symbols, before participants got insight into the task structure, *M* = 93.2, *CI* = [76.5, 103.9], had higher ratings than CC1 symbols, after participants got insight into the task structure, *M* = 52.1, *CI* = [35.4, 68.8], *t*(2548) = 7.60, *p* < .001. For CC3 symbols it was the other way around, before participants got insight into the task structure, CC3 symbols had lower ratings , *M* = 66.1, *CI* = [49.4, 82.9], than after they gained insight, *M* = 117.4, *CI* = [100.7, 134.2], *t*(2548) = -9.48, *p* < .001.

For *Long-Term Expected Valence Ratings*, the model was based on 2592 observations, *R^2^_marginal_* = 0.490, *R^2^_conditional_* = 0.570, *ICC_SUBJECT_ID_* = 0.141, *ICC_SYMBOL_ID_* = 0.020. There was a significant CUE_CONTINGENCYxINSIGHT interaction, *F*(2, 2548) = 110.5, *p* < .001, and two significant main effects: INSIGHT, *F*(1, 2548) = 11.3, *p* < .001, and CUE_CONTINGENCY, *F*(1, 2457) = 1279.5, *p* < .001. The interaction revealed that CC2 symbols before participants got insight into the task structure, *M* = -101.4, *CI* = [-121.9, -81.0] did not differ significantly from CC2 symbols after participants got insight into the task structure, *M* = -115.9, *CI* = [-136.34, -95.4], *t*(2548) = 2.46, *p* = .208. CC1 symbols, before participants got insight into the task structure, *M* = 97.4, *CI* = [76.9, 117.8], had significantly higher ratings than CC1 symbols, after participants got insight into the task structure, *M* = 26.1, *CI* = [5.68, 46.], *t*(2548) = 12.17, *p* < .001. For CC3 symbols, it was the other way around, before participants got insight into the task structure, CC3 symbols had lower ratings , *M* = 65.4, *CI* = [44.89, 85.9], than after they gained insight, *M* = 117.1, *CI* = [96.55, 137.6], *t*(2548) = -8.83, *p* < .001.

*Behavioral Adaption in the Questionnaire Block.* We computed a generalized mixed effect model with participants’ choice as binary dependent variable. The link function was logit and the distribution binomial. We included the interaction of INSIGHTxCUE_CONTINGENCY and the main effects of INSIGHT and CUE_CONTINGENCY as fixed effects in our model. This resulted in de following formula: CHOICE ~ INSIGHT*CUE_CONTINGENCY + (1+CUE_CONTINGENCY + BLOCK + CUE_CONTINGENCY:BLOCK|SUBJECT_ID). The model was based on 2592 observations, *R^2^_marginal_* = 0.258, *R^2^_conditional_* = 0.946, *ICC_SUBJECT_ID_* = 0.802. There was a significant INSIGHTxCUE_CONTINGENCY interaction, *X^2^(2, N = 36) = 10.58, p = .005,* and one significant main effects: CUE_CONTINGENCY, *X^2^(2, N = 36) = 15.12, p < .001*. The main effect INSIGHT, *X^2^(1, N = 36) = 3.66, p =* .056, did not reach significance. To clarify the interaction we did post-hoc comparisons, which revealed that in the first round CC3 symbols, *Prob.* = .928, *CI* = [0.849, 0.968]*,* had equally high gambling probabilities as CC1 symbols, *Prob.* = .942, *CI* = [0.861-0.977]*, z* = 0.423, *p* = 1.00. At the same time CC2 symbols had significantly lower gambling probabilities in the first round, *Prob.* = .322, *CI* = [0.134-0.594] compared to CC1 symbols*, z* = 4.579, *p* < .001, and compared to CC3 symbols, *z* = 4.752, *p* < .001. However, after participants received insight into the task structure CC1 Symbols, *Prob.* = .600, *CI* = [0.134-0.594], and CC2 symbols, *Prob.* < .001, *CI* = [<0.001-0.344], did not differ significantly from one another, *z* = 2.485, *p* = 0.194. Then, CC3 symbols, *Prob.* = .988, *CI* = [0.920-0.998], had significantly higher gambling probabilities than CC1 symbols, *z* = -3.232, *p* = 0.018, and CC2 symbols, *z* = -3.212, *p* = 0.020.
